# Strain-Rate-Dependent Tensile Behaviour and Viscoelastic Modelling of Kevlar^®^ 29 Plain-Woven Fabric for Ballistic Applications

**DOI:** 10.3390/polym17152097

**Published:** 2025-07-30

**Authors:** Kun Liu, Ying Feng, Bao Kang, Jie Song, Zhongxin Li, Zhilin Wu, Wei Zhang

**Affiliations:** 1 School of Mechanical Engineering, Nanjing University of Science and Technology, Nanjing 210094, Chinasj0501510127@126.com (J.S.); njustlzx@163.com (Z.L.); zhangweinjust@126.com (W.Z.); 2 School of Mechanical Engineering, Nanjing Institute of Technology, Nanjing 211112, China; 3 Unit 63856, People’s Liberation Army of China, Baicheng 137001, China; kangbao123@163.com

**Keywords:** Kevlar^®^, plain-woven, tensile mechanical properties, strain rate effect, constitutive model

## Abstract

Aramid fibre has become a critical material for individual soft body armour due to its lightweight nature and exceptional impact resistance. To investigate its energy absorption mechanism, quasi-static and dynamic tensile experiments were conducted on Kevlar^®^ 29 plain-woven fabric using a universal material testing machine and a Split Hopkinson Tensile Bar (SHTB) apparatus. Tensile mechanical responses were obtained under various strain rates. Fracture morphology was characterised using scanning electron microscopy (SEM) and ultra-depth three-dimensional microscopy, followed by an analysis of microstructural damage patterns. Considering the strain rate effect, a viscoelastic constitutive model was developed. The results indicate that the tensile mechanical properties of Kevlar^®^ 29 plain-woven fabric are strain-rate dependent. Tensile strength, elastic modulus, and toughness increase with strain rate, whereas fracture strain decreases. Under quasi-static loading, the fracture surface exhibits plastic flow, with slight axial splitting and tapered fibre ends, indicating ductile failure. In contrast, dynamic loading leads to pronounced axial splitting with reduced split depth, simultaneous rupture of fibre skin and core layers, and fibrillation phenomena, suggesting brittle fracture characteristics. The modified three-element viscoelastic constitutive model effectively captures the strain-rate effect and accurately describes the tensile behaviour of the plain-woven fabric across different strain rates. These findings provide valuable data support for research on ballistic mechanisms and the performance optimisation of protective materials.

## 1. Introduction

Ballistic vests, as essential personal protective equipment in modern warfare, play a crucial role in reducing combat casualties and enhancing individual survivability. Due to their superior strength-to-weight ratio and exceptional impact resistance, aramid fibres have been widely adopted as the primary material for soft armour systems [1,2]. Upon projectile penetration, aramid-based soft armour absorbs kinetic energy through multiple mechanisms, including fibre tensile fracture, fabric deformation, and interlayer delamination, with tensile-dominated failure serving as the principal mode of energy dissipation for ballistic protection [3]. Accordingly, investigation of the tensile mechanical properties of aramid materials holds significant military relevance for both elucidating ballistic mechanisms and optimising the protective performance of soft armour systems. Advancements in computational modeling have enabled the development of robust and highly realistic simulations to quantify both local and global damage in Kevlar^®^ armour subjected to ballistic penetration. These studies provide valuable insights through numerical investigations—particularly in determining the mechanical parameters required for constitutive law implementation—while also validating finite element (FE) models that accurately capture bullet penetration behavior and time-history responses. Numerical modeling of armour–penetrator interactions plays a crucial role in interpreting ballistic performance, necessitating predictive capabilities regarding material failure and stress wave propagation under dynamic loading conditions. Achieving such predictive accuracy depends on obtaining representative mechanical properties across a wide range of strain rates. Consequently, a thorough understanding of the constitutive and failure behavior of Kevlar^®^ 29 plain-woven fabric under tensile loading is essential for refining these models and enhancing their reliability.

In recent years, studies on the tensile mechanical properties of aramid materials and their woven fabrics have become a focal point in composite materials research. At the fabric level, researchers have developed corresponding constitutive models based on systematic experimentation. Shim et al. [4] conducted tensile mechanical tests on CT716 plain-woven aramid fabric, revealing significant strain-rate dependencies in its tensile strength, elastic modulus, and failure strain, and subsequently proposed a viscoelastic constitutive relationship to characterise this behaviour. Through combined experimental and numerical approaches, Naik and Stahlecker et al. [5,6] demonstrated a correlation between energy absorption mechanisms and both fabric architecture and fibre material properties in Kevlar^®^ 49 and Zylon^®^ fibre fabrics.

At the fibre and yarn scale, researchers have achieved deeper mechanistic insights. Cheng et al. [7] developed a specialised testing apparatus to analyse the nonlinear mechanical behaviour and stress-softening phenomena of Kevlar^®^ KM2 fibres. Using scanning electron microscopy (SEM), Sanborn et al. [8] elucidated fracture mechanisms of fibres subjected to varying strain rates at the microscopic scale. Through comparative studies, Tapie et al. [9] demonstrated that both T1040 Twaron^®^ virgin yarns and woven yarns exhibit characteristic strain-rate dependence—namely, increased strength and stiffness accompanied by reduced failure strain at higher loading rates. Wang and Xia [10] conducted tensile experiments across a wide strain-rate range (10^−4^ to 10^3^ s^−1^), revealing that the strength distribution of Kevlar^®^ 49 fibres follows Weibull statistical principles. Using a Split Hopkinson Tensile Bar (SHTB) apparatus, Zhu and Sun [11] observed ductile fracture characteristics in Kevlar^®^ fibres under high-strain-rate loading. Liu et al. [12,13] adopted a three-element viscoelastic model to characterise the dynamic response of Kevlar^®^ 49 yarns, with numerical simulations confirming the model’s applicability. Zhu et al. [14,15] systematically investigated scale effects on the mechanical properties of Kevlar^®^ fibres, identifying an inverse correlation between fibre strength and gauge length. Zhou et al. [16] established a strain-rate-dependent viscoelastic constitutive model, providing a theoretical framework for predicting the dynamic mechanical behaviour of aramid yarns.

Current research has significantly advanced the understanding of the tensile mechanical properties of Kevlar^®^, demonstrating the effectiveness of various characterisation techniques. However, most existing studies concentrate on the fibre and yarn scales, with limited emphasis on systematic investigations of fabric-level mechanical performance. Constitutive models applicable to woven fabrics remain underdeveloped, and the mechanisms governing multi-scale correlations in mechanical behaviour require further exploration.

This study investigates the quasi-static and dynamic tensile behaviour of Kevlar^®^ 29 plain-woven fabric through systematic experiments conducted using a universal material testing machine and an SHTB apparatus. Microscopic damage mechanisms were characterised via SEM and ultra-depth three-dimensional microscopy. A strain-rate-dependent viscoelastic constitutive model was developed to quantify the mechanical response. These findings provide essential data support for enhancing the ballistic protection performance in body armour systems.

## 2. Experimental Protocol

### 2.1. Specimen Preparation

**Figure 1:**
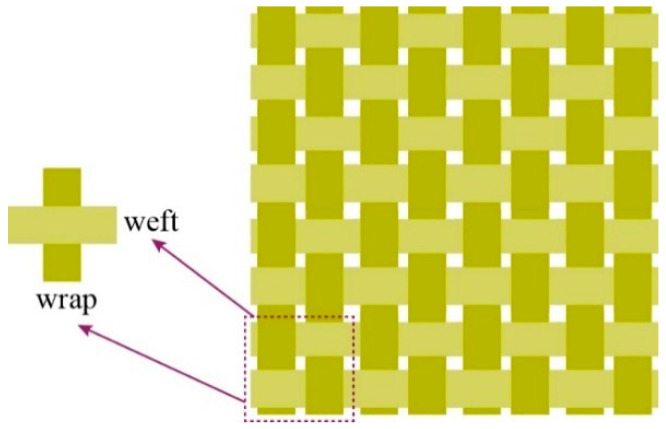
Schematic illustration of plain-woven Kevlar^®^ fabric architecture.

**Figure 2:**
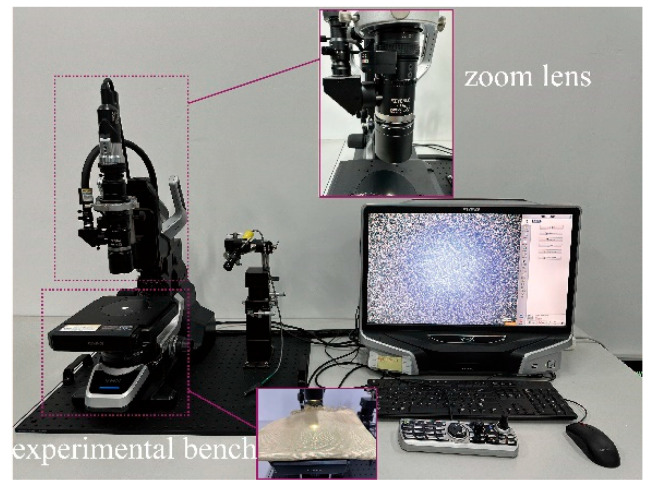
Ultra-depth optical microscopy system.

**Figure 3:**
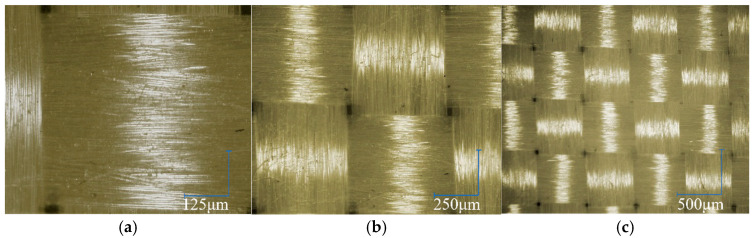
Micrographs of Kevlar^®^ plain-woven fabric: (**a**) 200 times magnification; (**b**) 100 times magnification; and (**c**) 50 times magnification.

The experimental study employed plain-woven Kevlar^®^ fabric manufactured by DuPont^TM^, as illustrated in Figure 1. The fabric features a characteristic 1/1 basket weave pattern, in which each warp yarn alternately passes over and under successive weft yarns. It exhibits equal warp and weft yarn densities of 9 yarns/cm. Detailed fabric parameters are summarised in Table 1. The macroscopic morphology of Kevlar^®^ plain-woven fabric was captured using an ultra-depth 3D microscopy system (KEYENCE Corporation, Osaka, Japan), as shown in Figure 2, with the corresponding micrograph presented in Figure 3. Figure 3 reveals that the Kevlar^®^ fiber bundles are not tightly packed, exhibiting discernible inter-bundle gaps. The cross-sectional area of Kevlar^®^ fiber bundles can be determined by the ratio of linear density to volumetric density, with the actual cross-sectional area of this aramid fiber bundle measuring approximately 7.7 × 10^−4^ cm^2^.

**Figure 4:**
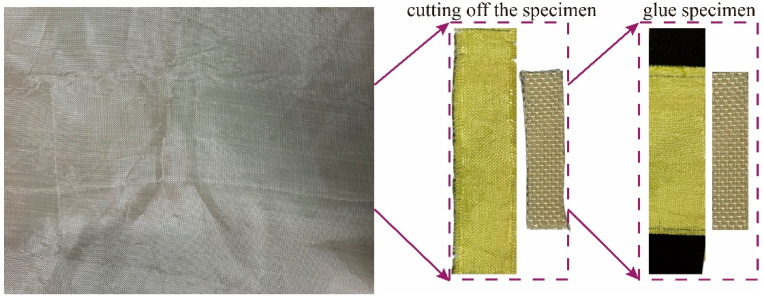
Fabrication process of Kevlar^®^ plain-woven fabric specimens.

**Figure 5:**
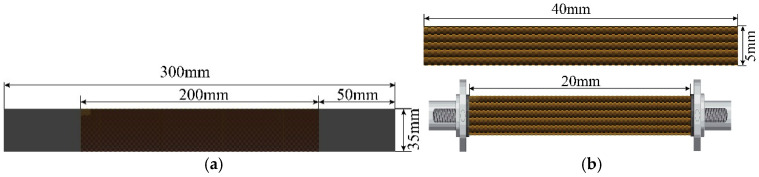
Schematic illustration of quasi-static and dynamic tensile specimen dimensions: (**a**) quasi-static tensile specimen; and (**b**) dynamic tensile specimen.

The quasi-static specimens were rectangular, with effective dimensions of 200 mm × 35 mm, comprising 28 yarns aligned along the loading direction. Both ends of each specimen were reinforced using 240-grit silicon carbide (SiC) paper bonded with epoxy resin and cured for over 24 h. The dynamic tensile specimens featured a rectangular gauge section measuring 20 mm × 5 mm, containing 5 yarns aligned in the loading direction. These specimens were directly mounted onto the fixtures using epoxy resin and cured for 24 h. The specimen preparation process is illustrated in Figure 4, and the detailed dimensions of both quasi-static and dynamic tensile specimens are illustrated in Figure 5.

### 2.2. Experimental Methodology and Procedures

#### 2.2.1. Quasi-Static Tensile Testing

**Figure 6:**
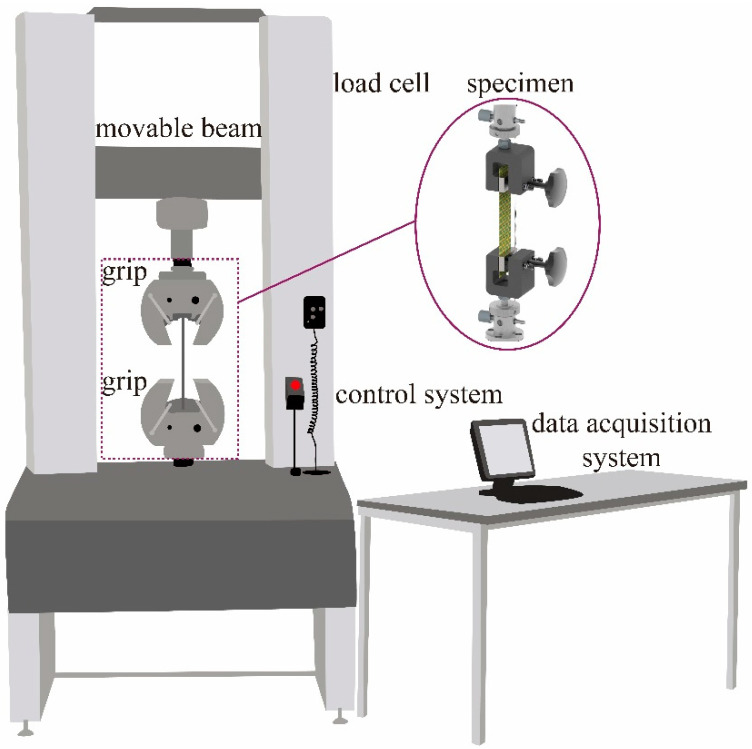
Principle of quasi-static tensile testing.

**Figure 7:**
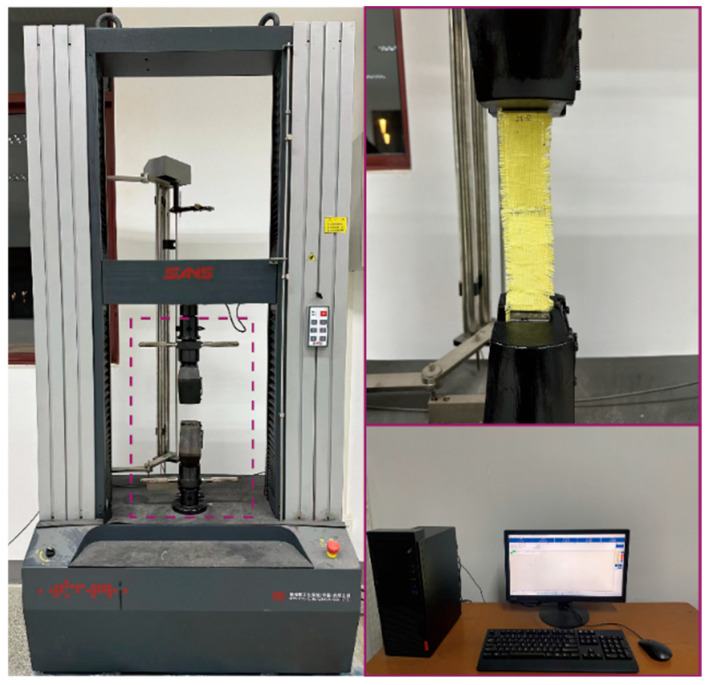
Universal testing machine system.

Uniaxial tensile tests were conducted on Kevlar^®^ plain-woven fabric specimens along the warp direction using a universal material testing machine (CMT 5105, MTS) with a maximum load capacity of 100 kN. Displacement-controlled loading was applied, and the experimental setup is illustrated in Figure 6. The specimen axis was carefully aligned with the machine axis, and manual wedge grips were used to secure the specimens. A preload of 25 N was applied at both ends to ensure proper initial tension. Tests were performed at three displacement rates: 1.2, 70, and 300 mm/min, and continued until complete specimen failure. The universal testing machine configuration is illustrated in Figure 7. To minimise random error, each loading rate condition was repeated three times, with force and displacement data recorded synchronously throughout the experiments.

Based on the measured load and displacement data, the engineering stress *σ*, strain *ε*, and strain rate $\dot{\varepsilon }$ of the specimen can be calculated as follows [17]:

(1) $$\sigma =\frac{F}{A_{s}},$$

(2) $$\varepsilon =\frac{\Delta l_{s}}{l_{s}},$$

where *F* is the loading force, *A*_s_ is the initial cross-sectional area of the specimen, *σ* is the stress, *ε* is the strain, *l*_s_ is the gauge length of the specimen, ∆*l*_s_ is the elongation of the gauge length.

The tensile modulus of the plain-woven fabric can be determined from the initial linear segment of the stress–strain curve and is expressed as follows:

(3) $$E=\frac{\Delta \sigma }{\Delta \varepsilon },$$

where *E* is the tensile modulus, ∆*σ* is the stress increment in the linear stage, and ∆*ε* is the strain increment in the linear stage.

#### 2.2.2. Dynamic Tensile Testing

**Figure 8:**
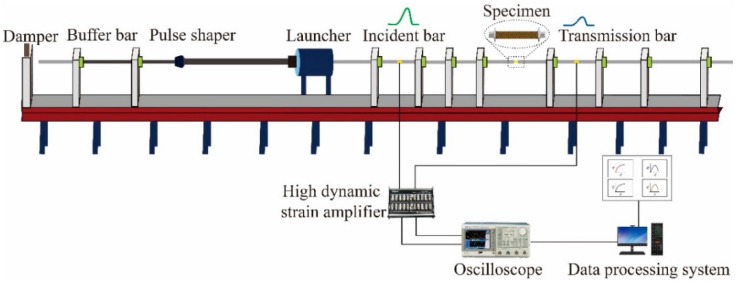
Schematic diagram of the SHTB experimental setup.

Dynamic tensile experiments along the meridional direction were performed using an SHTB setup with a bar diameter of 14.5 mm. The apparatus primarily consists of an incident bar, flange, transmission bar, launching device, and absorption device. A schematic of the setup is illustrated in Figure 8.

To extend the pulse rise time, smooth the waveform, and eliminate high-frequency oscillations, four square copper pulse shapers were evenly affixed to the impact surface of the flange at the end of the incident bar. The bars were fabricated from 60Si2MnA spring steel, with the material parameters provided in Table 2. The striker bar measured 200 mm in length, the incident bar 3000 mm, and the transmission bar 1500 mm.

**Figure 9:**
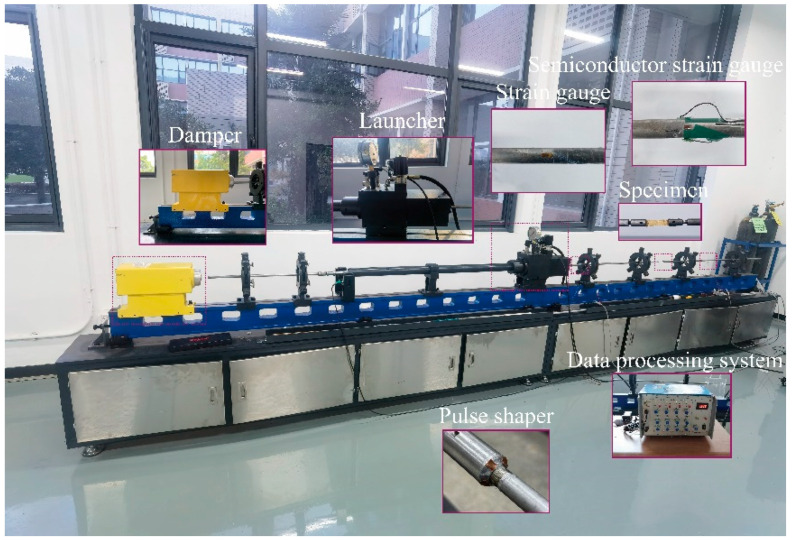
SHTB experimental system.

For strain measurement, a resistance strain gauge (sensitivity: 2.1, resistance: 120 Ω; model: BF120-5AA, Hanzhong Precision Electrical Co., Ltd., Hanzhong, China) was affixed to the midpoint of the incident bar, whereas a semiconductor strain gauge with a high signal-to-noise ratio (sensitivity: 100, resistance: 120 Ω; model: TP-3.8-120, Bengbu Kechuang Sensor Co., Ltd., Bengbu, China) was mounted on the transmission bar. These gauges, in conjunction with a dynamic strain amplifier, were used to capture the strain response characteristics of the specimen. The specimen fixture was threaded to both the incident and transmission bars, ensuring precise alignment of the specimen centre with the bar axis. The SHTB experimental system is illustrated in Figure 9.

Based on the strain signals acquired from the strain gauges mounted on the incident and transmission bars via the data acquisition system, the strain rate, strain, and stress of the specimen were determined using one-dimensional stress wave theory, expressed as follows [18,19,20,21,22]:

(4) $$\dot{\varepsilon }\left(t\right)=-\frac{2c_{0}}{l_{s}}\varepsilon_{r}\left(t\right),$$

(5) $$\varepsilon \left(t\right)=-\frac{2c_{0}}{l_{s}}{\int_{0}^{t}\varepsilon }_{r}\left(t\right)dt,$$

and

(6) $$\sigma \left(t\right)=\frac{E_{0}A_{0}}{A_{s}}\varepsilon_{t}\left(t\right),$$

where *c*_0_ represents the elastic wave velocity in the tensile bars, *E*_0_ denotes the elastic modulus of the bar material, *A*_0_ is the cross-sectional area of the bars, and *ε*_r_(*t*) and *ε*_t_(*t*) correspond to the strain signals associated with the reflected and transmitted waves in the bars, respectively.

## 3. Experimental Results and Analysis

### 3.1. Quasi-Static Tensile Test Results

**Figure 10:**
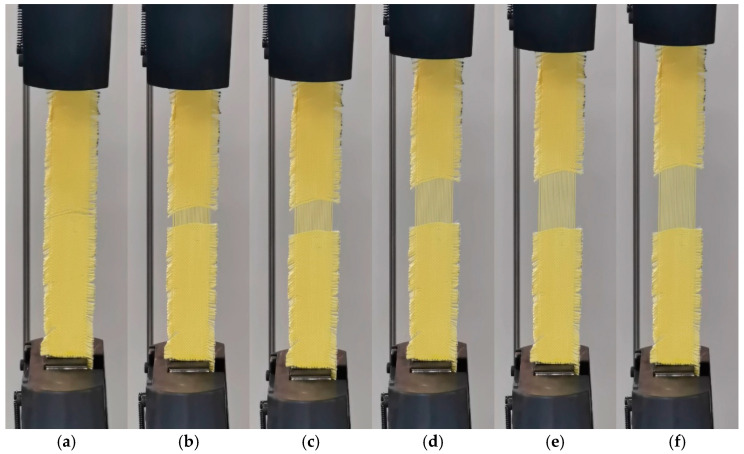
The quasi-static tensile deformation process of Kevlar^®^ plain-woven fabric at a tensile rate of 20 mm/min: (**a**) *t* = 31 s; (**b**) *t* = 62 s; (**c**) *t* = 93 s; (**d**) *t* = 124 s; (**e**) *t* = 155 s; and (**f**) *t* = 186 s.

Figure 10 illustrates the quasi-static tensile deformation process of Kevlar^®^ plain-woven fabric at a tensile rate of 20 mm/min. As illustrated in the figure, under tensile loading, fibre separation initially occurs in the central region of the specimen, forming a visible notch. As load increases, the inter-fibre spacing progressively expands until the peak load is reached, at which point partial fibre breakage occurs along the loading direction. Subsequently, the load gradually decreases as extensive failure develops in the central region, accompanied by the emergence of fibrous filaments.

**Figure 11:**
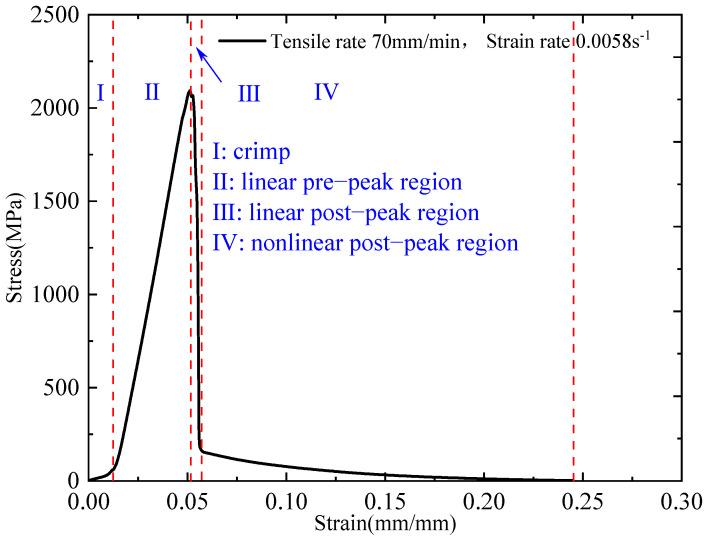
Quasi-static stress–strain curve at 70 mm/min tensile rate.

Figure 11 presents the stress–strain curve of the plain-woven fabric at a tensile rate of 70 mm/min. The curve can be divided into four distinct stages: the fibre crimp region (I), the linear pre-peak region (II), the linear post-peak region (III), and the nonlinear post-peak region (IV).

In Stage I, due to the inherent crimping of fabric fibres introduced during manufacturing, tensile deformation along the loading direction is constrained, resulting in a nonlinear stress–strain response with a gradual increase in stress. This stage concludes at a stress of 0.061 MPa and a strain of 0.0123 mm/mm.

Stage II is characterised by the progressive straightening of the fabric under continued loading. A linear relationship between stress and strain is observed, indicative of elastic deformation without evidence of plasticity or damage.

Stage III begins at the peak stress of 2.1 GPa, where fibre failure initiates along the loading direction. This leads to a decline in load-bearing capacity. The stress decreases linearly, ending at a strain of 0.057 mm/mm and a stress of 0.162 GPa, corresponding to widespread fibre failure.

In Stage IV, stress decreases nonlinearly as the strain increases to approximately 0.25 mm/mm. This behaviour reflects the progressive detachment and pull-out of failed fibres from the fabric, ultimately reducing the stress to zero.

**Figure 12:**
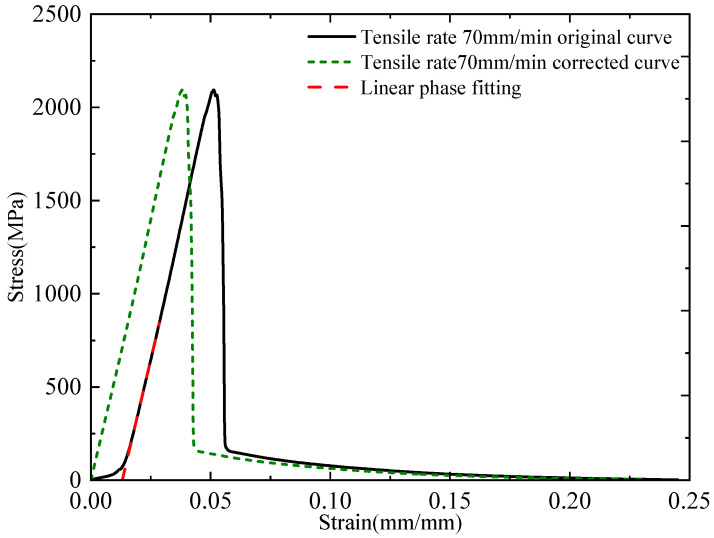
Corrected stress–strain curve at 70 mm/min tensile rate.

Notably, Stage I corresponds to the straightening of the fabric’s naturally crimped morphology, which contributes minimal strain relative to the overall failure strain and is therefore considered negligible in mechanical analysis. To more accurately evaluate the quasi-static mechanical properties of the plain-woven fabric, the nonlinear portion corresponding to Stage I was excluded from the corrected stress–strain curve, as illustrated in Figure 12.

**Figure 13:**
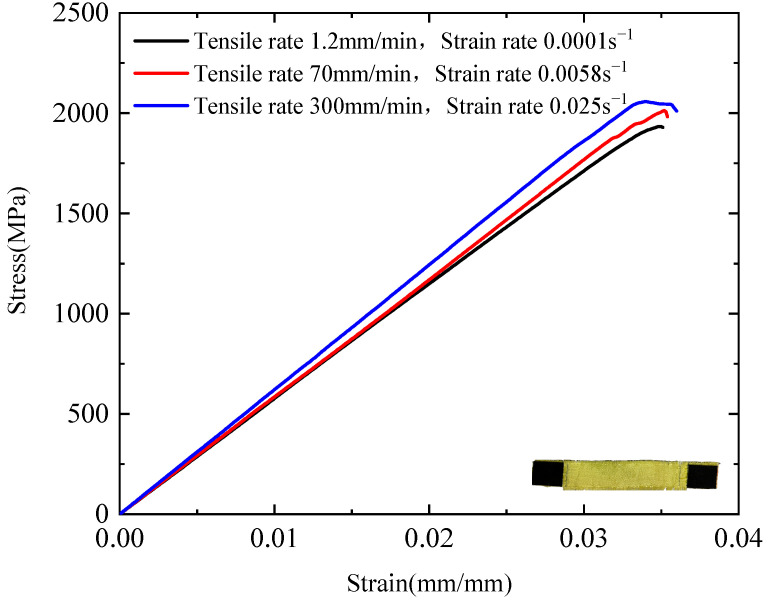
Quasi-static stress–strain curves at different strain rates.

Figure 13 presents the quasi-static stress–strain curves of the plain-woven fabric under different strain rates. At tensile rates of 1.2, 70, and 300 mm/min (corresponding to strain rates of 0.001, 0.0058, and 0.025 s^−1^, respectively), the tensile strength values are 1.94, 2.01, and 2.06 GPa; the failure strains are 0.035, 0.034, and 0.033 mm/mm; the tensile moduli are 57.4, 58.5, and 62.0 GPa; the toughness values are 35.2, 36.6, and 39.9 MJ/m^3^; and the elongations at break are 4.7%, 4.6%, and 4.5%., respectively. These results demonstrate that the plain-woven fabric exhibits clear strain-rate dependence under quasi-static tensile loading. Specifically, tensile strength, tensile modulus, and toughness increase with rising strain rate, whereas failure strain and elongation at break decrease accordingly.

### 3.2. Dynamic Tensile Test Results

**Figure 14:**
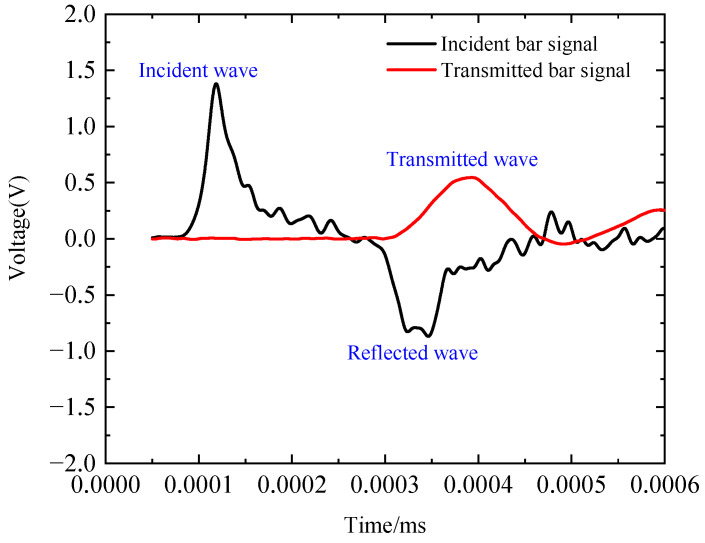
Waveform characteristics at 420 s^−1^ strain rate.

**Figure 15:**
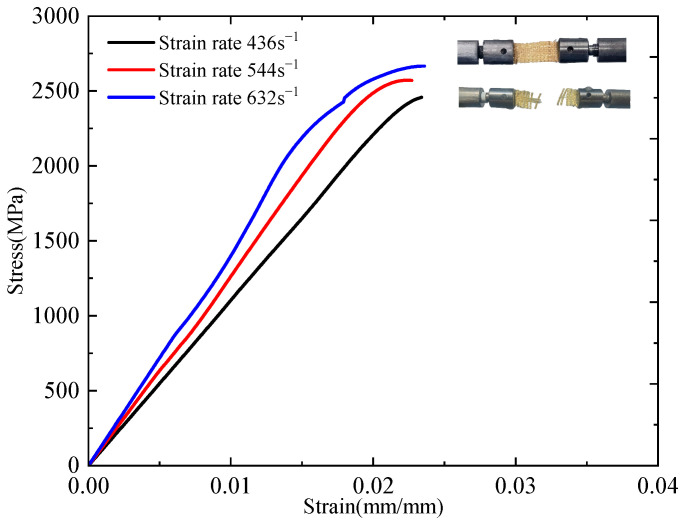
Dynamic stress–strain curves under different strain rates.

The incident, reflected, and transmitted waveforms at a strain rate of 420 s^−1^ are illustrated in Figure 14. By zeroing the signals from the incident and transmitted bars and aligning the wave fronts of the transmitted and reflected waves, the strain rate, strain, and stress of the dynamic tensile specimen were calculated using Equations (5)–(7). Figure 15 presents the dynamic stress–strain curves at different strain rates. The mechanical properties exhibit a clear strain-rate dependence, with increasing strain rates (436, 544, and 632 s^−1^), resulting in systematic variations in material performance. Specifically, the tensile strength increases from 2.5 GPa to 2.6 GPa and further to 2.7 GPa, whereas the failure strain slightly decreases from 0.0234 to 0.0224 and 0.0222 mm/mm. Concurrently, the tensile modulus shows a progressive increase from 108 GPa to 127 GPa and 138 GPa, and the toughness improves from 30.1 MJ/m^3^ to 32.3 MJ/m^3^ and 37.7 MJ/m^3^, indicating enhanced energy absorption at higher strain rates. These results clearly demonstrate that under dynamic tensile loading, the plain-woven fabric exhibits significant strain-rate dependence: tensile strength, elastic modulus, and toughness increase with rising strain rate, whereas failure strain decreases. Compared to quasi-static loading conditions, the fabric displays brittle behaviour, with specimens undergoing complete fracture [16].

### 3.3. Microstructural Damage Characterisation

**Figure 16:**
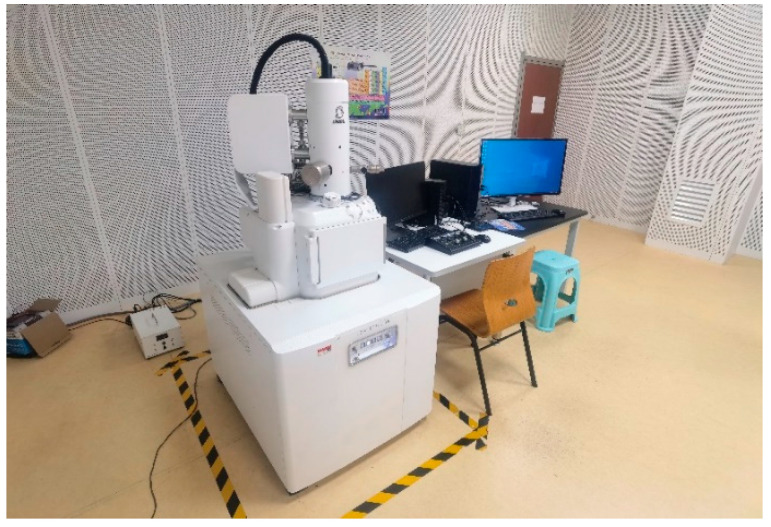
SEM system.

**Figure 17:**
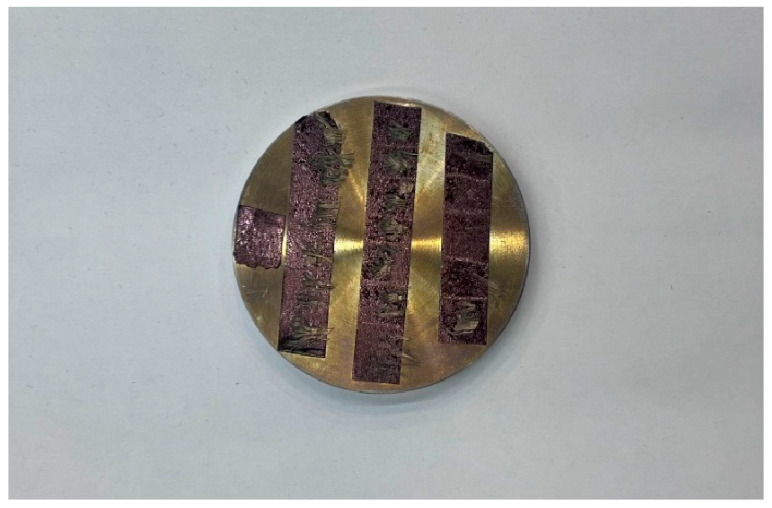
Gold-sputtered specimen preparation.

The microscopic damage and fracture characteristics of Kevlar^®^ plain-woven fabric fibres were examined using SEM (JSM-IT500HR, JEOL Ltd., Tokyo, Japan) and a three-dimensional ultra-depth microscopy system. The SEM apparatus and gold-sputtered specimens are illustrated in Figure 16 and Figure 17, respectively.

**Figure 18:**
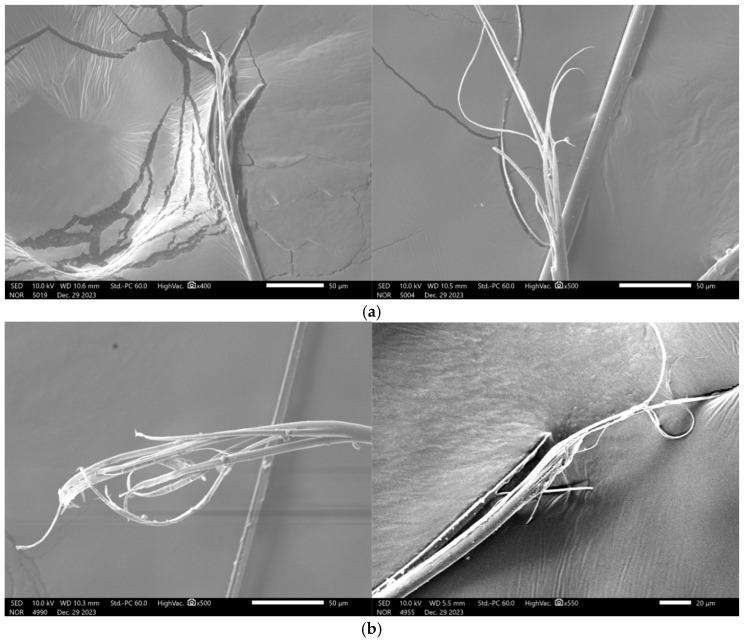
SEM images of quasi-static and dynamic tensile fracture surfaces: (**a**) quasi-static tensile specimen; and (**b**) dynamic tensile specimen.

Figure 18 presents SEM images of the tensile fracture surfaces of the specimens. The fabric fibres exhibit a distinctive skin–core structure, consisting of an outer thin skin layer and an inner core layer. Under tensile loading, fracture of the skin layer causes the inner core to lose constraint and disintegrate, resulting in fibre axial splitting, fibrillation, and plastic flow. Following a tensile fracture, numerous microfibrils are generated along the original fibre axis.

Under quasi-static conditions, the fracture surfaces show evidence of plastic flow, with slight axial splitting and tapered fibre ends—features characteristic of ductile fracture. In contrast, under dynamic tensile loading, the fracture ends appear sharply tapered. As the strain rate increases, fibres exhibit more pronounced axial splitting with reduced splitting depth. Simultaneous fracture of both the skin and core layers is observed on some fibre fracture surfaces, accompanied by fibrillation phenomena, indicating brittle fracture behaviour.

**Figure 19:**
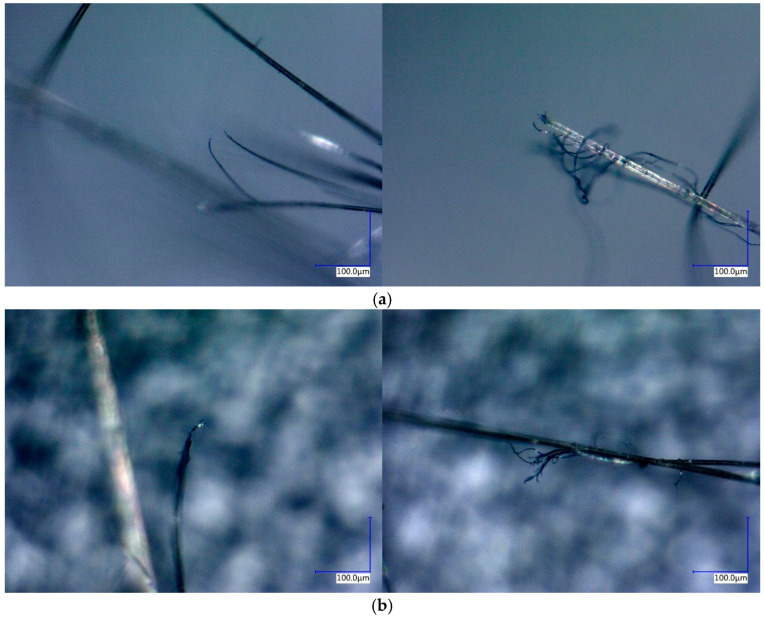
Optical micrograph of quasi-static and dynamic tensile fracture surfaces: (**a**) quasi-static tensile specimen; and (**b**) dynamic tensile specimen.

Under tensile loading, interactions between fibres weaken, resulting in slippage and tearing that give rise to dendritic fracture structures. Stress concentration at weak fibre points leads to rapid fracture, often accompanied by a rise in localised temperature, which manifests as scorching or carbonisation. The fracture surface exhibits pronounced roughness, affecting light reflection and causing scattering and darkening along the broken edges. Figure 19 shows a microscopic image of the tensile fracture surface. Intact fibres appear yellow, whereas fractured regions appear black, with visible branching phenomena.

## 4. Constitutive Model

### 4.1. Three-Element Viscoelastic Constitutive Model

**Figure 20:**
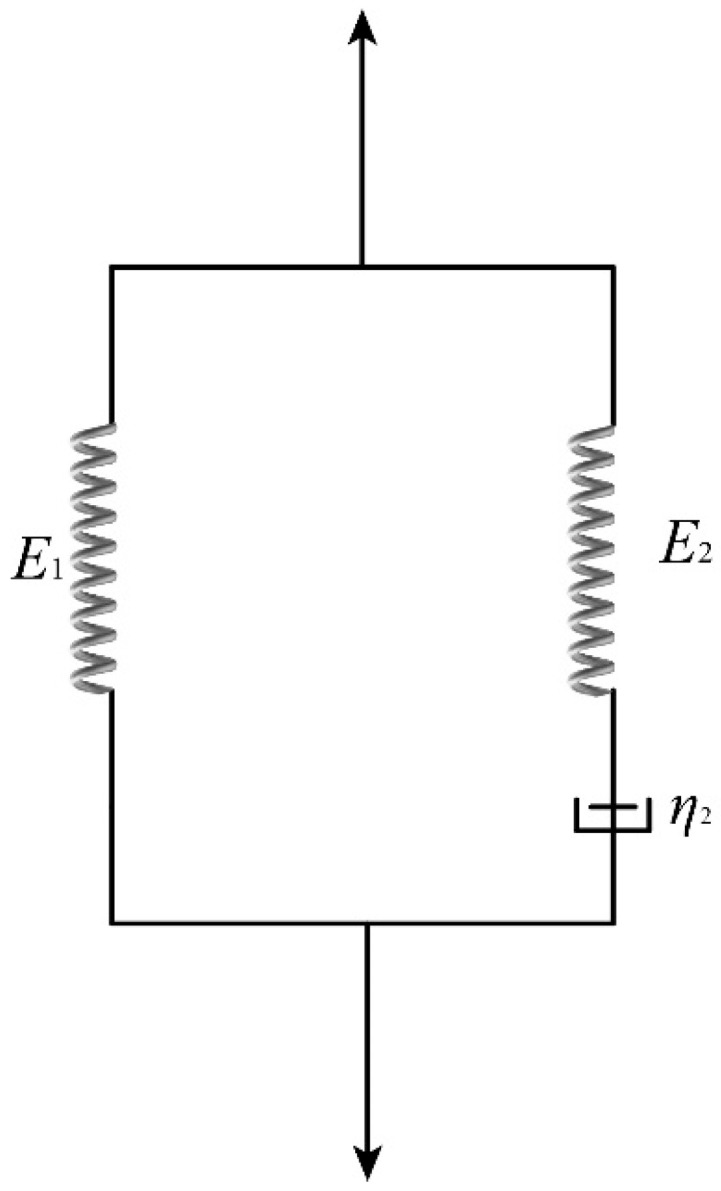
Three-element viscoelastic constitutive model.

Analysis of the quasi-static and dynamic stress–strain curves of Kevlar^®^ plain-woven fabric reveals its viscoelastic and strain-rate-dependent behaviour. A three-element viscoelastic constitutive model was employed to characterise its mechanical properties. The model comprises a single elastic element connected in parallel with a Maxwell body, as illustrated in Figure 20 [23,24].

The constitutive equation can be expressed as follows:

(7) $$\sigma \left(\varepsilon \right)=E_{1}\varepsilon +\eta_{2}\dot{\varepsilon }\left(1-e^{-\frac{E_{2}\varepsilon }{\eta_{2}\dot{\varepsilon }}}\right),$$

where *E*_1_ and *E*_2_ denote the elastic constants of the spring elements, whereas *η*_2_ represents the viscous damping coefficient.

Under low-strain-rate loading conditions, Equation (8) can be simplified as follows:

(8) $$\sigma \left(\varepsilon \right)=E_{1}\varepsilon .$$

The constitutive model parameters were determined through global fitting using the least squares method, as summarised in Table 3.

**Figure 21:**
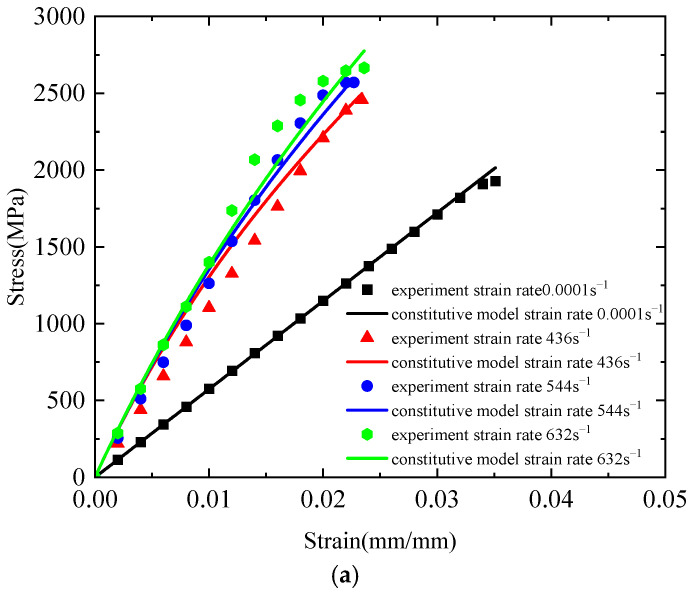

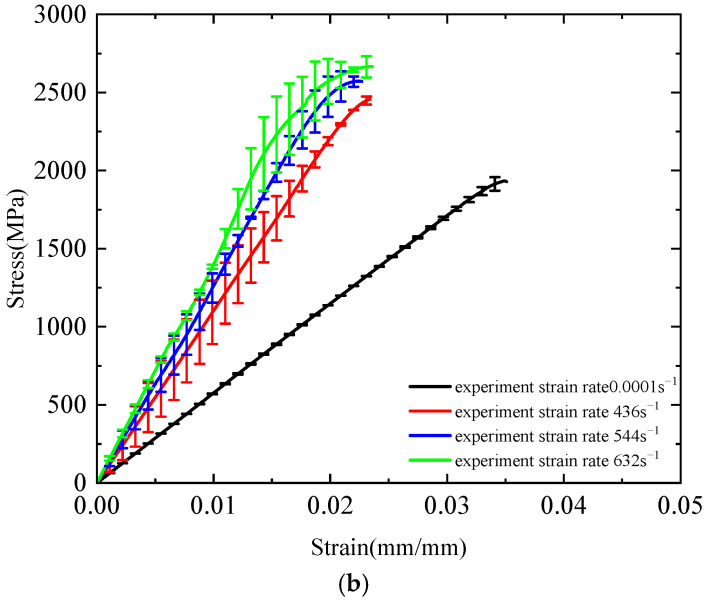
Comparison between theoretical and experimental curves of the three-element viscoelastic constitutive model: (**a**) theoretical curve and experimental curve; and (**b**) error bar curve.

Figure 21a presents a comparison between the theoretical curve derived from the three-element viscoelastic constitutive model and the corresponding experimental stress–strain curves. Under quasi-static tensile conditions, the theoretical curve closely aligns with the experimental data, demonstrating excellent fitting performance with a correlation coefficient of 0.999. However, as the strain rate increases, the correlation gradually decreases. At a strain rate of 436 s^−1^, significant discrepancies arise between the theoretical and experimental curves during the elastic stage, whereas good agreement is maintained in the nonlinear stage, resulting in a correlation coefficient of 0.964. At 544 s^−1^, the model provides a good fit in the elastic stage but shows noticeable deviations in the nonlinear region, with a correlation coefficient of 0.973. At an even higher strain rate of 632 s^−1^, considerable deviations are observed throughout the curve despite a correlation coefficient of 0.979. These results indicate that, although the three-element viscoelastic constitutive model effectively captures the strain-rate dependence of Kevlar^®^ plain-woven fabric, it exhibits limitations in accurately representing the tensile mechanical response across a wide range of strain rates. Figure 21b shows the error bars between the theoretical and experimental values of the three-element viscoelastic constitutive model. As can be seen from Figure 21b, at strain rates of 0.0001, 436, 544, and 632 s^−1^, the maximum errors between the theoretical and experimental values are 86.19, 205.6279, 135.4986, and 345.0859 MPa, respectively.

### 4.2. Modified Three-Element Viscoelastic Constitutive Model

The three-element viscoelastic constitutive model was modified to address the aforementioned limitations. As indicated in the stress–strain curves of the plain-woven fabric, the tensile modulus increases with increasing strain rate. Accordingly, a strain-rate-dependent term was introduced into the elastic component of the three-element viscoelastic model. The modified constitutive model can be expressed as follows:

(9) $$\sigma \left(\varepsilon \right)=\left(E_{1}+E_{3}\dot{\varepsilon }\right)\varepsilon +\eta_{2}\dot{\varepsilon }\left(1-e^{-\frac{E_{2}\varepsilon }{\eta_{2}\dot{\varepsilon }}}\right),$$

where *E*_3_ denotes the elastic constant of the spring element.

The parameters of the modified constitutive model obtained via least squares fitting according to Equation (9) are listed in Table 4.

**Figure 22:**
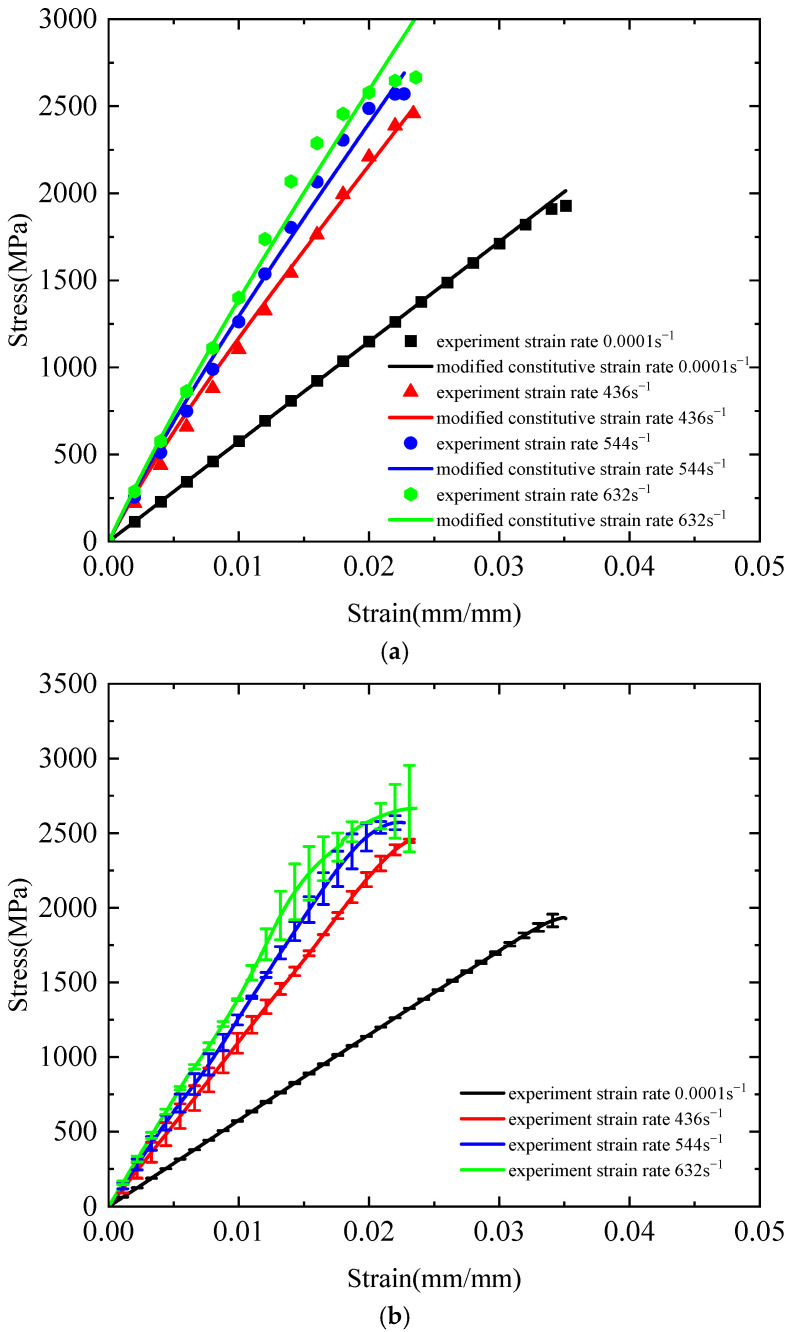
Comparison between theoretical and experimental curves of the modified three-element viscoelastic constitutive model: (**a**) theoretical curve and experimental curve; and (**b**) error bar curve.

Figure 22a presents a comparison between the theoretical curves derived from the modified three-element viscoelastic constitutive model and the experimental data. As illustrated in the figure, at low strain rates, the modified model exhibits excellent agreement with the experimental results, with a correlation coefficient of 0.999. Under dynamic tensile conditions, the fitted curves also demonstrate strong consistency with the experimental data. At a strain rate of 436 s^−1^, the correlation coefficient reaches 0.994; while at 544 s^−1^, only minor deviations are observed between the theoretical and experimental curves, yielding a correlation coefficient of 0.996. At 632 s^−1^, the theoretical predictions closely match the experimental measurements, with a correlation coefficient of 0.983. Figure 22b presents the error bar curve comparing the theoretical and experimental values of the modified three-element viscoelastic constitutive model. It can be observed that at strain rates of 0.0001, 436, 544, and 632 s^−1^, the maximum discrepancies between theoretical and experimental values are 85.1, 83.1889, 119.8466, and 243.9279 MPa, respectively.

**Figure 23:**
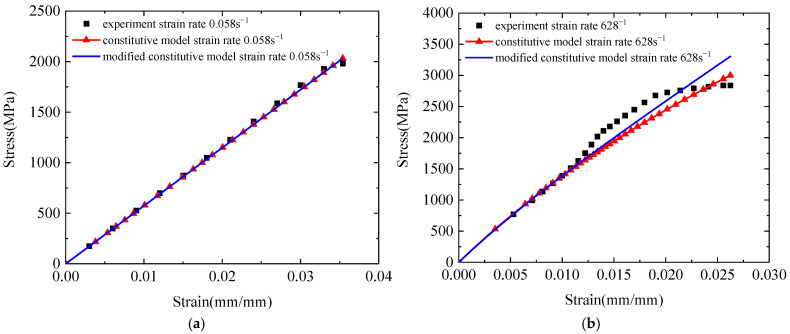
Comparison of stress–strain curves between the three-element viscoelastic constitutive model and its modified version: (**a**) $\dot{\varepsilon }$ = 0.058 s^−1^; and (**b**) $\dot{\varepsilon }$ = 628 s^−1^.

For further validation, Figure 23 compares the predicted curves from both the original three-element viscoelastic model and the modified constitutive model with experimental data at strain rates of 0.058 s^−1^ and 628 s^−1^. The results demonstrate the enhanced applicability of the modified model. These findings confirm that the modified three-element viscoelastic constitutive model effectively captures the strain-rate effects in Kevlar^®^ plain-woven fabrics and accurately describes their tensile mechanical behaviour across a wide range of strain rates.

## 5. Conclusions

A study on the energy absorption mechanism of Kevlar^®^ 29 plain-woven fabric was conducted through quasi-static and dynamic tensile experiments. Fracture morphology was examined using SEM and ultra-depth three-dimensional microscopy. Microscopic damage patterns were analysed, and a viscoelastic constitutive model was developed to incorporate strain-rate effects. The main conclusions are as follows:

1. Under quasi-static tensile loading, the stress–strain response of the fabric can be divided into four distinct stages: the fibre crimp region, linear pre-peak region, linear post-peak region, and nonlinear post-peak region. The fabric exhibits clear strain-rate sensitivity, with tensile strength, Young’s modulus, and toughness increasing as the strain rate increases, whereas fracture strain and elongation at break decrease accordingly.
2. Under dynamic tensile loading, the plain-woven fabric demonstrates pronounced strain-rate dependence. As the strain rate increases, tensile strength, elastic modulus, and toughness increase, whereas fracture strain decreases. Compared to quasi-static conditions, the fabric exhibits brittle fracture behaviour, resulting in complete specimen failure.
3. Under quasi-static loading, fracture surfaces display evidence of plastic flow, with slight axial splitting and tapered fibre ends, features characteristic of ductile fracture. The ductile fracture behavior is associated with a necking mechanism, wherein localised plastic flow is achieved through dynamic hydrogen bond reorganization and fibrillar slippage of molecular chains, thereby retarding crack propagation. In contrast, dynamic loading produces sharply tapered fracture ends. At higher strain rates, significant axial splitting occurs with reduced splitting depth. Some fibres exhibit simultaneous rupture of both the surface and core layers, accompanied by fibrillation phenomena, indicating brittle fracture behaviour. Following the tensile failure, numerous microfibrils are observed along the fibre axis, along with localised temperature rise, resulting in charring or carbonisation. The brittle fracture behavior follows a crazing mechanism, wherein the hydrogen bond network and molecular chains cannot reorganize under rapid loading, leading to rapid nucleation and propagation of microcracks at stress concentration sites (e.g., grain boundaries or defects).
4. The modified three-element viscoelastic constitutive model effectively captures the strain-rate-dependent mechanical behaviour of plain-woven Kevlar^®^ fabrics and accurately describes their tensile response across a range of strain rates. These findings provide valuable data for advancing ballistic protection research and optimising the performance of protective materials.

## Figures and Tables

**Table 1:** Kevlar^®^ plain weave fabric material parameters.

Areal Density	Cross-Sectional Area	Linear Density	Yarn Count	Volumetric Density	Fibre Type	Manufacturer
/(g·m^−2^)	/(cm^2^)	/(g·m^−1^)	/D	/(g·cm^−3^)		
200	7.7 × 10^−4^	0.11	1000	1.44	Kevlar^®^ 29	DuPont^TM^

**Table 2:** Material parameters of tensile bars.

Wave Impedance	Wave Velocity	Poisson’s Ratio	Elastic Modulus	Density	Material
/(kg·m^−2^·s^−1^)	/(m·s^−1^)		/(GPa)	/(g·cm^−3^)	
4.07 × 10^7^	5.18 × 10^3^	0.3	1.44	7.85	60Si2MnA Spring steel (Huoxin, Luoyang, China)

**Table 3:** Parameters of the three-element viscoelastic constitutive model.

*η*_2_/(MPa·s)	*E*_2_/(GPa)	*E*_1_/(GPa)
3.23	103.11	57.4

**Table 4:** Parameters of the modified three-element viscoelastic constitutive model.

*η*_2_/(MPa·s)	*E*_3_/(MPa)	*E*_2_/(GPa)	*E*_1_/(GPa)
0.59	87.5	45.6	57.4

## Data Availability

The original contributions presented in the study are included in the article, further inquiries can be directed to the corresponding author.
